# Balancing Needs in Publishing With Undergraduate and Graduate Students at Doctoral Degree-Granting Universities

**DOI:** 10.3389/fpsyg.2019.00295

**Published:** 2019-03-01

**Authors:** Rebecca A. Lundwall, Cooper B. Hodges, Allison D. Kotter

**Affiliations:** Lundwall Lab, Psychology Department, Brigham Young University, Provo, UT, United States

**Keywords:** publishing, mentoring, writing experience, undergraduates, graduate students

## Introduction

Professors at doctoral-degree granting universities tend to focus on publishing with graduate students more than with undergraduates. While we argue that publishing with undergraduates is worthwhile, we first want to point to organizational structures that contribute to the focus on graduate students. First, the hierarchical structure of doctoral universities can make publishing with undergraduates more difficult. Although it is often possible to delegate mentoring of undergraduates to graduate students, faculty have primary responsibility for mentoring graduate students (Espinoza-Herold and Gonzalez, 2007; Ynalvez et al., 2014). Direct faculty mentoring of graduate students is necessary because success in obtaining postdoctoral positions, faculty appointments, and research-related employment is highly dependent on publishing with mentors while in graduate school (Hartley and Betts, 2009; Casanave, 2010). Second, compared with undergraduate programs, graduate programs tend to provide more field-specific knowledge, greater depth of study, and increased focus on conducting research (Mangematin, 2000; Austin, 2002; Hakala, 2009; Northwest Commission on Colleges Universities., 2018). A graduate student's knowledge of the subfield can make publishing with graduate students less time consuming. Third, faculty at high research activity universities are under considerable pressure to publish frequently and in high-impact journals (Nir and Zilberstein-Levy, 2006; Burks and Chumchal, 2009; Rizzo Parse, 2009; Everett and Earp, 2015), which makes publishing without students tempting. Nevertheless, publishing together can be rewarding for faculty, graduate students, and undergraduate students.

## Rewards of Publishing With Undergraduate Students

Despite the understandable pressures to focus on graduate students, publishing with undergraduate researchers can be uniquely rewarding for faculty members (Kardash, 2000; Burks and Chumchal, 2009; Styles, 2009; Hartley, 2014). For example, engaging with undergraduate researchers sometimes reminds faculty of the curiosity they had as beginning researchers (Styles, 2009; Shanahan et al., 2015; Bathgate and Schunn, 2017). Several faculty researchers have noted that it is rewarding to train a new generation of researchers. Passing science on to undergraduate student researchers by writing and publishing with them plays a fundamental role in bringing new minds through the ranks (Burks and Chumchal, 2009; Lopatto, 2010; Rogers et al., 2012; Urias et al., 2012).

It is also true that the undergraduates who work directly with a faculty member tend to rate these experiences as highly beneficial (Hunter et al., 2007; Lopatto, 2010; Shanahan et al., 2015; Heiden, 2018). Research experience allows undergraduates to determine if they like research. Since writing and publishing are difficult, a student who can publish has strong evidence of their interest in research, persistence, and writing ability. In addition, writing about research can help undergraduates feel pride in contributing to scientific knowledge. Should the student apply to graduate school, the qualities they gain by doing and writing about research will be highly valued by admissions committees and graduate school mentors (Huss et al., 2002; Kierniesky, 2005; Burks and Chumchal, 2009).

## Suggestions for Publishing With Undergraduate Students

Although publishing with undergraduates can be challenging, we (a lab director, graduate student, and former undergraduate research assistant) share strategies we have used while mentoring 130 undergraduate and six graduate student researchers over the past 5 years, with approximately 25 students participating in the lab at a time. We developed these suggestions with input from current and former undergraduate and graduate students. In short, we suggest that faculty provide a mentoring structure, recruit effectively, prepare research assistants, teach writing skills, set clear expectations, and employ graduate students wisely.

### Provide a Mentoring Structure

As might be imagined, in a large and productive lab, keeping track of everyone's projects and assignments can be challenging. To ease the faculty member's load, we suggest using a modified hierarchical structure for mentoring (Wilson et al., 2012; Newman et al., 2015; Shanahan et al., 2015). This structure usually involves graduate students mentoring undergraduates, which allows undergraduates to get many of their questions answered while graduate students gain experience in providing mentoring. We modify this approach by suggesting that faculty also interact directly with undergraduate researchers in key areas of impact. For example, we work in small groups with motivated and trained undergraduate research assistants to complete conference presentations and writing projects. We meet weekly or bi-weekly to discuss issues and make writing assignments (including for the faculty member). This approach has the benefit of maintaining faculty–undergraduate interaction while also saving some faculty time. I (RAL) have successfully used this approach to co-author with students on approximately 70% of my publications (see vita at https://fhssfaculty.byu.edu/FacultyPage?id=lbecky64).

### Recruit Effectively

There are other aspects of publishing with both graduate and undergraduate students that faculty researchers might want to consider. First, it is important to consider how to go about recruiting research assistants. Whatever skills a faculty member wants a research assistant to have must have been acquired or taught in previous research experiences, in courses, or by the faculty member or lab staff. Faculty members should also decide if they want to wait for students to seek them out or if they prefer actively recruiting. If the faculty member opts for actively recruiting, some possibilities include announcing openings in their courses or emailing all current psychology majors. A course on preparing for graduate school, if offered, will be a promising source for recruiting students interested in research and publication. We have also had good experiences recruiting undergraduates who have recently completed a research methods course. If you do recruit, be careful not to overlook women and minorities, who are less likely to approach professors to ask about working in their labs (Chan, 2008; Hurtado et al., 2011). We have recruited more diverse research assistants by advertising that, “all students interested in research, including women and minorities, should apply.” Even if you do not actively recruit, consider providing a statement on your faculty webpage indicating what interested undergraduates should do and providing a link to any application you would like them to complete (see the application at https://cogdevelopment.byu.edu/Pages/home.aspx). Having instructions and applications ready can simplify the process when students express interest in to working in your lab.

### Prepare Research Assistants

If getting undergraduates excited about research motivates a faculty member or graduate student, they should consider the needs of undergraduates and what will help them prepare for research careers. As mentioned above, undergraduates are often missing background in the faculty researcher's field, which can put more pressure on faculty at doctoral universities. During lab meetings and small-group meetings, we have provided general advice, including how to impress a potential reference letter-writer, deal with writer's block, develop time-management skills, and give 3-min summaries of their research projects. Undergraduate researchers may also need help understanding how researchers develop research ideas and designs to test them. These activities often seem mysterious to undergraduates and need to be made explicit (Wei and Woodin, 2011; Hampden-Thompson and Sundaram, 2013). Making research practices more explicit can prevent frustration and encourage undergraduates to become more involved and excited about research. The same is true for how to approach writing, for example, an introduction section. Making transparent whatever steps you take in these tasks (regardless of whether your processes are like other researchers'), can help undergraduates understand one way they can approach these tasks.

### Teach Writing Skills

Undergraduates often lack the experience in writing that graduate students have (Burks and Chumchal, 2009; Shanahan et al., 2015) and many do not learn about discipline-specific writing until they take an advanced writing course (Emerson et al., 2006; Grzyb et al., 2018). To help develop discipline-specific writing skills, we have had undergraduate researchers develop mini-research proposals and empirical reports (one page) on subsets of projects from the lab and present these at lab meetings. These follow the Introduction, Method, Results, and Discussion format (with Anticipated Results and Conclusion replacing the last two elements for proposals). Alternatively, students may choose to present a simplified (one- or two-page) version of a faculty member's papers (i.e., a high-level summary of each section in an article) and discuss these in lab meetings. These activities may also help undergraduate researchers become more comfortable talking about research in a specific lab. While I acknowledge that other faculty members may engage in similar activities, a thorough review of all university library databases indicates that neither of these ideas has been discussed in the literature. Addressing the effectiveness of this approach in comparison to other approaches would be a good next step toward establishing best practices in writing with undergraduates.

### Set Clear Expectations

If you frequently publish with undergraduate students, word will get out. Undergraduates are frequently told in graduate school preparation courses that they need to present posters and publish manuscripts to stand out as applicants for graduate school. The advice they have received can make them eager to publish but not necessarily eager to engage with research more broadly. Therefore, we suggest that faculty members be clear about their expectations for involvement in other aspects of the lab and what qualifies for co-authorship on papers from the lab. If a student is not participating in research for course credit or as an employee (where expectations are often in writing), then a mentoring agreement can be helpful to clarify expectations. The mentoring agreement that we use was developed by our university general counsel in collaboration with our department (see https://cogdevelopment.byu.edu/Pages/home.aspx). The mentoring agreement defines mentoring as primarily for the benefit of the student, indicates data ownership, describes the right for either mentee or mentor to terminate the relationship with or without cause, and lists activities the mentee might be able to participate in (including, if desired, contributing to a manuscript). The mentee must acknowledge that there is no guarantee of publication or future employment. As advised by others, at least once a semester, we tell students what qualifies for credit in acknowledgments, co-authorship, and primary authorship (APA Science Student Council, 2006; Burks and Chumchal, 2009). Clear, consistent communication in these ways minimizes misunderstandings and makes students aware of the opportunities available to them.

### Employ Graduate Students Wisely

For researchers at doctoral degree-granting institutions, it is also important to decide how to employ graduate students wisely. Although graduate students going into academia will need publications (Mangematin, 2000; Jalongo et al., 2014; Pennycook and Thompson, 2018), they may also need experience supervising undergraduates, who can help the graduate students on their research projects and publications. Undergraduates may find it easier to ask a graduate student some questions. Almost half of undergraduates in courses I (RAL) teach indicate that they feel more comfortable approaching a graduate student than a faculty member for help. However, they may feel neglected if the faculty member does not also take an interest in their work and get to know them. Therefore, it seems best to share responsibilities with graduate students in mentoring undergraduates.

## Conclusion

Faculty members at doctoral universities are sometimes hesitant to get involved in writing projects with undergraduate researchers. Undergraduates might have little background in the research areas of a faculty member and—due to less experience with writing—are also more likely than graduate students to need their writing extensively edited by the faculty member. Without minimizing the needs of graduate students for faculty mentoring or the challenges involved in mentoring undergraduate researchers, as a faculty member, I (RAL) nonetheless enjoy instilling excitement for research in undergraduates. As lab leaders, we try to remember that undergraduate and graduate students alike are often motivated by curiosity—which is a worthwhile attitude to encourage a new generation of researchers (Chin and Osborne, 2008; Gottfried et al., 2016; Bathgate and Schunn, 2017). Attending to different needs between student groups and involving graduate students in mentoring undergraduates can make researching and writing with undergraduates more feasible so faculty can support curiosity and still balance their own publishing needs (Haslam and Laham, 2009; Stanley et al., 2017).

## Author Contributions

All authors listed have made a substantial, direct and intellectual contribution to the work, and approved it for publication.

### Conflict of Interest Statement

The authors declare that the research was conducted in the absence of any commercial or financial relationships that could be construed as a potential conflict of interest.
